# A Comparison of Frontal Theta Activity During Shooting among Biathletes and Cross-Country Skiers before and after Vigorous Exercise

**DOI:** 10.1371/journal.pone.0150461

**Published:** 2016-03-16

**Authors:** Harri Luchsinger, Øyvind Sandbakk, Michael Schubert, Gertjan Ettema, Jochen Baumeister

**Affiliations:** 1 Centre for Elite Sports Research, Department of Neuroscience, Norwegian University of Science and Technology, Trondheim, Norway; 2 Institute of Sports Medicine, Department Exercise & Health, University of Paderborn, Paderborn, Germany; University of L'Aquila, ITALY

## Abstract

**Background:**

Previous studies using electroencephalography (EEG) to monitor brain activity have linked higher frontal theta activity to more focused attention and superior performance in goal-directed precision tasks. In biathlon, shooting performance requires focused attention after high-intensity cross-country skiing.

**Purpose:**

To compare biathletes (serving as experts) and cross-country skiers (novices) and examine the effect of vigorous exercise on frontal theta activity during shooting.

**Methods:**

EEG frontal theta (4–7 Hz) activity was compared between nine biathletes and eight cross-country skiers at comparable skiing performance levels who fired 100 shots on a 5-m indoor shooting range in quiescent condition followed by 20 shots after each of five 6-min high-intensity roller skiing sessions in the skating technique on a treadmill.

**Results:**

Biathletes hit 80±14% and 81±10% before and after the roller skiing sessions, respectively. For the cross-country skiers these values were significantly lower than for the biathletes and amounted to 39±13% and 44±11% (p<0.01). Biathletes had on average 6% higher frontal theta activity during shooting as compared to cross-country skiers (F1,15 = 4.82, p = 0.044), but no significant effect of vigorous exercise on frontal theta activity in either of the two groups were found (F1,15 = 0.14, p = 0.72).

**Conclusions:**

Biathletes had significantly higher frontal theta activity than cross-country skiers during shooting, indicating higher focused attention in biathletes. Vigorous exercise did not decrease shooting performance or frontal theta activity during shooting in biathletes and cross-country skiers.

## Introduction

The ability to focus on adequate sensory information related to performance is one of the key elements for success in elite sports. This ability is often referred to as focused attention, and several studies have shown a positive relationship between brain correlates of focused attention and performance in goal-directed precision tasks [1–3]. In the Olympic sport of Biathlon, the biathletes ski loops of 2.5–5 km in the skating technique carrying a rifle and perform five shoots of rifle shooting between loops [4]. The sport, hence, is performed at high exercise intensity while cross-country skiing [5] and requires focused attention during shooting [1]. The shooting task is highly complex, demanding coordination of visual input, postural balance and timing the contraction of the index finger at trigger pull [6].

Brain activity measured by electroencephalography (EEG) is regarded a reliable method for recording scalp potential fluctuations as a non-invasive measure of cortical oscillations from underlying neural assemblies [7]. EEG has grown in popularity among sports scientists over the last two decades due to improvements in both hardware and software, thereby minimizing the contamination of the EEG-signal by artifacts during human movement tasks. A well-known component in this context is the frontal theta frequency, which has a range of 4–7 Hz, and is most prominent over midline fronto-central electrodes. Source modeling attempts have localized the frontal theta activity to the anterior cingulate cortex (ACC) or the medial frontal cortex [3,8,9]. The ACC as a possible source to frontal theta is consistent with results from other neuroimaging studies [10], from studying monkeys [11] and a brain lesion study [12]. This anatomical region is thought to be part of the human executive attentional system involved in the mechanisms for monitoring and modulating sensory input [13]. Frontal theta activity has also been found to increase with working memory load [3,9,14], indicating a possible role of theta oscillations in working memory maintenance.

The working memory theory explains how we temporarily store, process and act on sensory information [3, 14, 15]. In this concept, higher theta activity in frontal electrodes was related to higher levels of focused attention and enhanced performance in golf [2] and during rifle shooting [1, 16]. When comparing experts and novice shooters, Doppelmayr et al. [1] found a significantly higher frontal theta activity in experts in the interval from -1 s to -0.5 seconds before trigger pull. Furthermore, higher frontal theta activity was found during the aiming period in expert sharpshooters [16] and in brain states during putting in golfers [2] compared to novices. Following exhaustive exercise, a significant decrease in performance was accompanied by a decrease in frontal theta activity during a knee-angle reproduction task in 12 male participants [17], suggesting that exhaustive exercise decreases the amount of attentional resources. This could be related to central fatigue or to a change in the sensory feedback from locally fatigued muscles causing higher demands for cortical processing of that feedback and thereby less frontal theta activity.

While differences between experts and novices in frontal theta activity appears related to focused attention, other frequency bands, such as alpha (8–13 Hz) and beta frequencies (13–30 Hz) [16,18,19,20] have been related to other factors involved in performance. For example, Haufler et al. [16] documented more low-frequency alpha (9 Hz) activity in the left temporal region in novices compared to experts during the aiming period of rifle shooting. In addition, Del Percio et al. [20] documented lower general cortical activation among elite air pistol shooters during the aiming period. This was suggested to be related to more selective activation of neurological pathways.

To the best of our knowledge, no previous study has concurrently compared experts and novices and, at the same time, examined the effects of vigorous exercise on frontal theta activity. Due to its stationary nature, thus minimizing movement artefacts, shooting in biathlon serves as a goal-directed precision task that can be examined with EEG. Therefore, the purpose of this study was to compare frontal theta activity between biathletes (serving as experts) and cross-country skiers (novices) during shooting and to examine the effects of vigorous exercise between the two groups. The hypotheses were that biathletes would show higher frontal theta activity during shooting as compared to cross-country skiers, and that vigorous exercise would decrease frontal theta activity during shooting to a greater extent among cross-country skiers than biathletes.

## Methods

### Participants

Nine biathletes and eight cross-country skiers participated in this experimental study. The characteristics of the participants are documented in Table 1. The biathletes were recruited from a biathlon team in central Norway and inclusion criteria was set to at least five years of biathlon experience and two or more weekly shooting trainings. The cross-country skiers were recruited as control group as they matched biathletes in physical capacity and had no experience with rifle shooting. Before providing their written consent to participate, athletes were fully informed about the nature of the study, and that they could withdraw from the experiment at any time without giving an explanation. The study was registered at Norwegian Social Science Data Services and the Regional Committee of Medical and Health Research Ethics in Central Norway gave permission to actuate the study.

**Table 1:** The characteristics of the nine biathletes and eight cross-country skiers participating in this study (mean ± SD).

	Biathletes	Cross-country skiers
**Gender**	7F + 2M	4F + 4M
**Age (yrs)**	21 ± 2	25 ± 1
**Body mass (kg)**	64.4 ± 7.5	67.9 ± 7.6
**Body height (cm)**	175 ± 10	177 ± 10
**Shooting experience (yrs)**	9 ± 2	0 ± 0

F, female; M, male

### Overall design

This cross-sectional study aimed to compare brain activity during shooting in biathlon in a rested state and after exhausting exercise between biathletes (serving as experts) and cross-country skiers (novices). The EEG frontal theta band between 4–7 Hz was compared between nine biathletes and eight cross-country skiers at comparable skiing performance levels when firing 100 shots on a 5 m indoor shooting range in quiescence followed by 20 shots after each of five 6-min vigorous roller skiing sessions in the skating technique on a treadmill.

### Instruments and materials

An EEG-cap (QuickCap, Compumedics, USA) connected to a NuAmps amplifier (Compumedics Neuroscan, USA) sampled EEG-data at 1000 Hz/22 bit from 32 electrodes placed on the scalp in accordance to the standard 10–20 system [21].The amplifier, that weighed 0.57 kg, was carried in a backpack. A Scatt Laser Shooter Training System including a Scatt biathlon electronic target (Model: SBT-5) and an optical sensor (Model: OS-02), together with the Scatt Biathlon software version 1.0.25 (Scatt Biathlon, Russia), recorded and provided instant feedback of performance by the target turning green on successful shots. The Scatt Biathlon shooting system and the sighting of the rifle was calibrated for the 5-m shooting range using the calibration process provided by the Scatt system. All participants used the same biathlon rifle (Anschutz Fortner sprint) with a mass of 3.7 kg. The loading handle of the rifle was connected to a switch that left a trigger in the EEG signals and thus synchronized the EEG-data with the shooting data. The roller skiing intervals were performed at 5% inclination on a 5 x 3 m motor-driven treadmill (Forcelink B.V., Culemborg, The Netherlands). Heart rate (HR) was recorded with a Garmin Forerunner 610 (Garmin Ltd., Olathe, KS) HR-monitor. Swenor skating roller skis, with resistance “2” wheels, were used and the participants used self-selected poles. An A4 sheet with a Borg scale [22] from 6–20 was placed such that it was readily available for recording participants’ Rate of Perceived Exertion (RPE). Participants wore shorts and t-shirt or similar indoor training clothes suited for physical exercise in 18 degrees Celsius.

### Procedures

**Fig 1:**
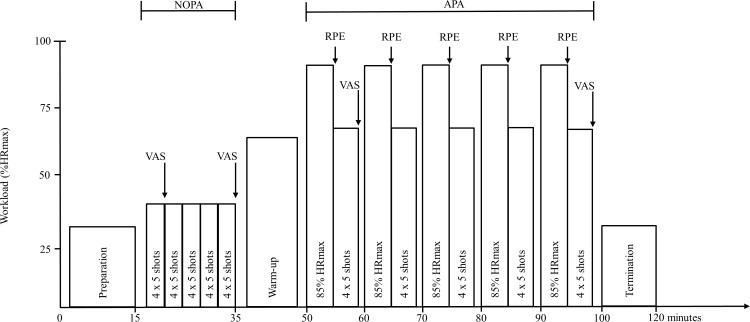
Illustration of the experimental protocol used in this study where nine biathletes (experts) and eight cross-country skiers (novices) performed 100 shots in rest (NOPA-condition) and after 5 x 6 minutes of vigorous roller ski skating intervals (APA-condition). Workload is displayed as a percent of the maximal heart rate (%HRmax). A signal-test where the participants were lying supine for five minutes was conducted after preparing the EEG-system to ensure stable contact with the electroencephalography-system and heart rate monitor (preparation). Electroencephalography, shooting performance and heart rate were measured during shooting in NOPA and APA. Self-reported concentration level (VAS) was rated after 20 and 100 shots in both conditions. After each interval, participants reported their rate of perceived exertion (RPE) on a Borg scale.

All participants had a session in the lab preceding the experiment to familiarize the lab setting and cross-country skiers were taught the basics of a standing biathlon shooting position. The experimental protocol is presented in Fig 1. Initially, the QuickCap was prepared with the Neuroscan QuickCell system and electrolyte, including a mid-forehead EEG ground electrode and linked earlobes used as average reference ((A1+A2)/2). Thereafter, impedance testing and visual inspection was done to ensure high quality of the EEG data and stable contact with the HR-monitor and the EEG-system. After preparing, the participants were lying supine for five minutes to quantify the resting state (REST). Subsequent to these five minutes, the EEG-signals were once again visually inspected for quality when participants were standing in shooting position before the participants fired 100 shots in five blocks of 4 x 5 shots in a “no physical activity” (NOPA) condition. A 30-second recovery between blocks was performed. The participants were instructed to hit as many targets as possible in their own self-chosen pace except for a compulsory extra second of aiming after trigger pull to make sure that it was possible to measure brain activity also after the trigger pull. This was followed by a 10-min warm-up while roller ski skating at a 5% inclination using a self-chosen technique on the treadmill with increasing intensity until the participants reached 80% of their self-reported maximal heart rate (HRmax) after 7–8 min. The last two minutes of the warm-up were performed at low intensity, followed by a two minutes break. During the 5 x 6 min interval performed with the Gear 3 technique with 5% inclination, their speeds were set individually at approximately 85–90% of HRmax estimated from their HR during the warm-up protocol. After each interval, the participants took their skis and poles off, stepped off the treadmill and walked 4 metres to the shooting range to pick up the rifle and start shooting. Twenty shots, in blocks of 4 x 5 shots after each interval, provided 100 shots in the “after physical activity” (APA) condition.

Participants had the opportunity to drink water before each interval. HR, RPE and self-reported concentration using a Visual Analogue Scale (VAS) were measured at specific times during the experiment (Fig 1) to validate the change in metabolic work as a result of the exercise, and to control for possible changes in these variables during both NOPA and APA-conditions.

### Exercise intensity measurements

HR was averaged over 10-second periods directly before the first shot and directly after the last shot in each shooting block. In addition, HR was also measured at 3:00 min and 5:30 min during each of the intervals. After each interval, participants reported their RPE, and the self-reported concentration level was obtained after 20 and 100 shots in both conditions on a 10 cm VAS scale.

### EEG processing

EEG-data was processed using the EEGlab-toolbox [23] for Matlab 2013b (Mathworks, Natick, United States). Initially, EEG resting state (REST) was quantified from continuously measured EEG data while lying in a dark room with both eyes closed [2]. Here, theta frequency (4–7 Hz) over all electrode locations was determined by applying a Fast Fourier Transformation for frequency power analysis to ensure the comparability between biathletes and cross-country skier groups. EEG-data from APA and NOPA conditions were epoched in the interval from 2 seconds before to 1 second after trigger pull (EVENT). The EVENT epoch was further divided into six shorter epochs (each lasting 0.5 second), and the average frontal theta activity during epoch 1–4 is defined as AIM. A finite impulse response band pass filter from 0.5 to 40 Hz removed 50 Hz line noise [24] and signals were re-referenced to the average of all electrodes. The raw signals were down-sampled to 250 Hz. An experienced electroencephalographer identified and–if present—deleted contaminated EEG channels before an adaptive mixed model independent component analysis (AMICA) was used to remove stereotypical artifacts such as eye-blinks from the raw signals [25]. Thus, only artifact-free epochs were included in the further analyses. The average amount of epochs after artifact rejection were 74% for the NOPA condition and 63% for the APA condition. A Fast Fourier Transform was applied for frequency-power analysis. Spectral power for theta frequencies between 4–7 Hz in the FZ electrode was analysed and referred to as frontal theta activity. The absolute activity values (log10(μV2), hereafter reported as μV2) for theta frequencies in the FZ electrode were log transformed to ensure a normal distribution [26]. One male biathlete was excluded from the analysis due to bad data.

### Statistical analysis

All sets of data were found to be normally distributed and means ± standard deviations are presented. An independent t-test was applied to analyse frontal theta differences between groups in REST. A two-way analysis of variance for repeated measures was used to analyse group (biathletes versus cross-country skiers) and condition (NOPA versus APA) interactions on frontal theta activity, shooting performance and self-reported concentration level. Independent samples t-tests were used to analyse group differences in average HR during shooting, average HR during intervals, average speed during intervals, and average RPE. At last, a two-way analysis of covariance was used to analyse group (biathletes versus cross-country skiers) and condition (NOPA versus APA) interactions on frontal theta activity in AIM and epoch 5 with condition as covariate. Statistical significance was set at an alpha level of < 0.05. All statistical analyses were done in SPSS version 22 (IBM Corp., Armonk, NY, USA).

## Results

**Fig 2:**
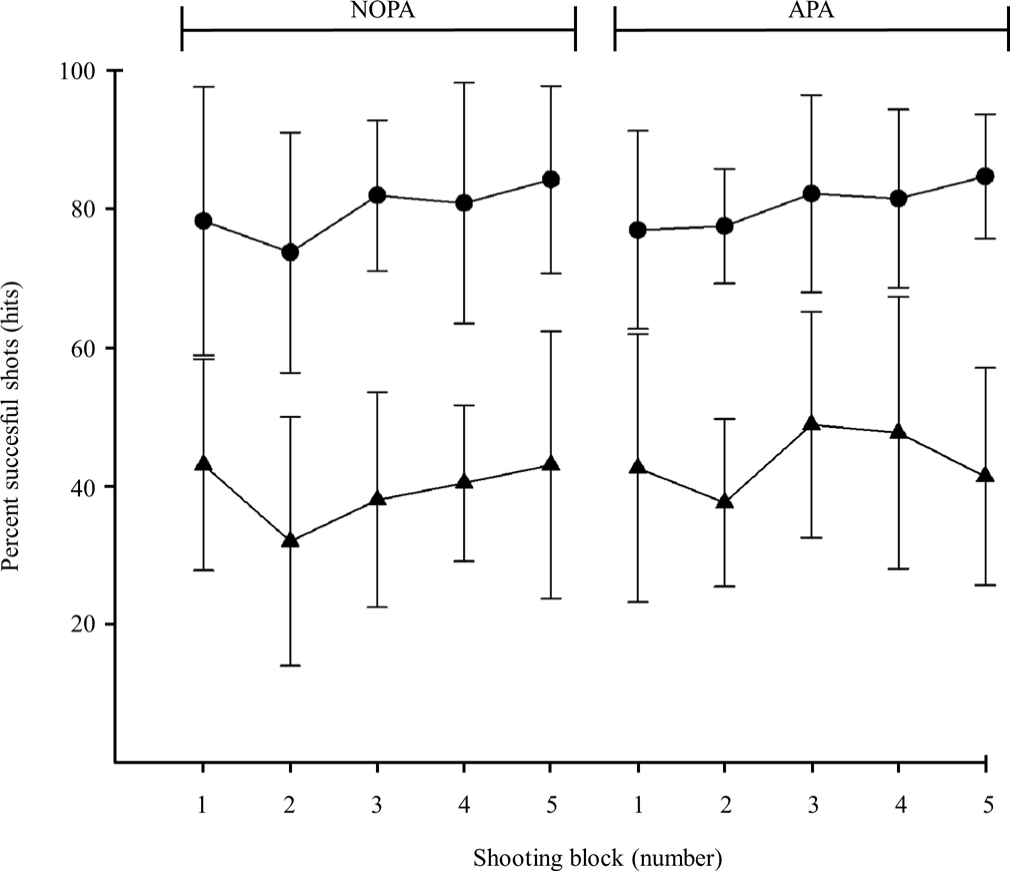
Shooting performance in percent of successful shots (hits) during blocks of 4 x 5 shots is illustrated for both the biathletes (circles) and cross-country skiers (triangles) in rested state (NOPA) and after 6-min vigorous roller ski skating exercise (APA) (mean and standard deviation).

Biathletes hit 80±14% and 81±10% before and after the roller skiing sessions, respectively. For the cross-country skiers these values amounted to 39±13% and 44±11% (Fig 2) which were significantly lower than in biathletes (main group effect: F1,15 = 48.3, p < 0.01). The vigorous-intensity intervals did not affect the shooting performance (condition interaction: F1,15 = 2.20, p = 0.16) nor did the intervals affect shooting performance differently among biathletes and cross-country skiers (group x condition interaction: F1,15 = 1.06, p = 0.32).

The HR during APA-shooting in biathletes and cross-country skiers was significantly higher than during NOPA-shooting (64 ± 4% vs. 39 ± 5% and 60 ± 5% vs. 40 ± 5% of HRmax for biathletes and cross-country skiers respectively; both p < 0.01). No significant group differences were found in HR during APA and NOPA, average HR during exercise intervals (88 ± 4% vs 86 ± 4% of HRmax), average speed during intervals (11.2 ± 2.2 vs 11.9 ± 1.3 km/t) or average RPE (14.4 ± 0.5 vs 14.5 ± 1.0). There was no significant group difference in the VAS scores (F1,14 = 0.19, p = 0.67), nor did the VAS-scores develop differently over time between biathletes compared to cross-country skiers (F3,42 = 2.30, p = 0.091).

**Fig 3:**
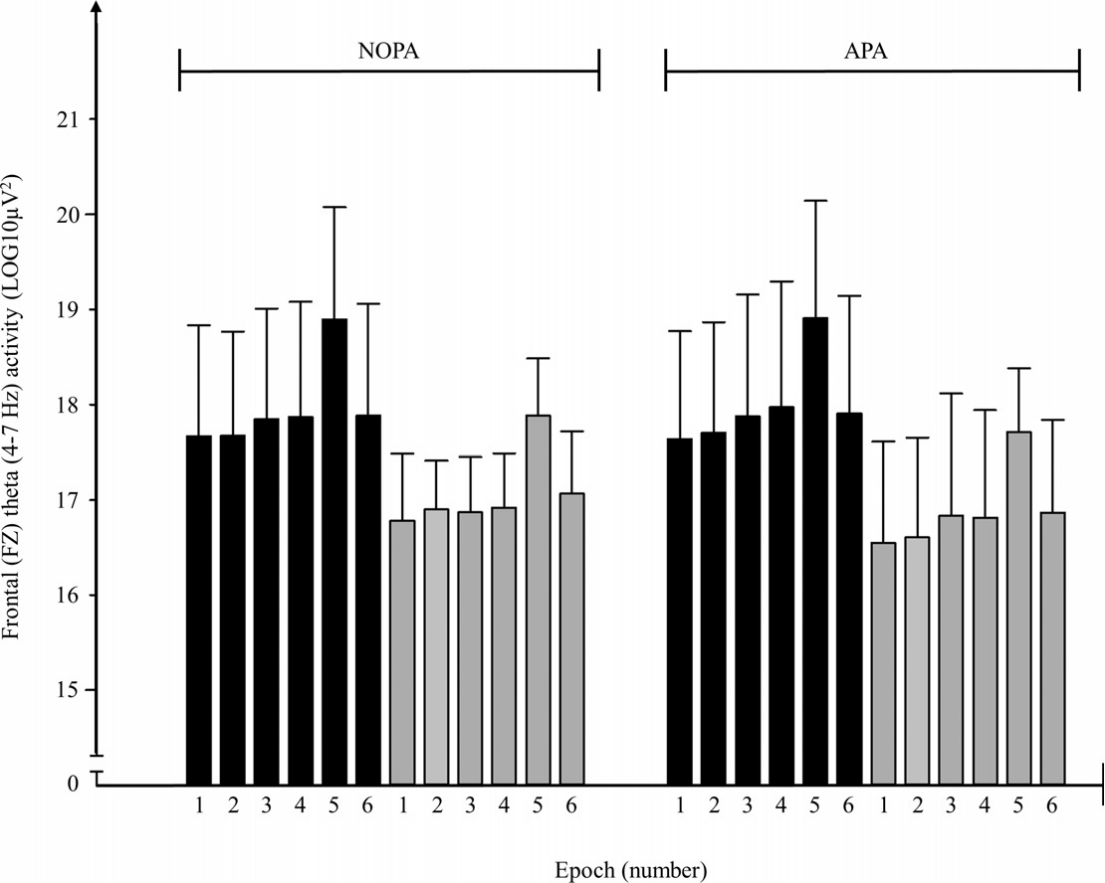
Theta (4–7 Hz) spectral activity measured in the frontal midline (FZ)—electrode on nine biathletes (black bars) and eight cross-country skiers (grey bars) during shooting in a rested state (NOPA) and after vigorous roller ski skating exercise (APA) (mean and standard deviation). Brain activity was analysed in the interval from 2 seconds before the trigger pull to 1 second after the trigger pull and divided into six 0.5-second epochs for further analysis

Frontal theta spectral power did not differ between biathletes and cross-country skiers in REST (t,15 = -0.593; p = 0.563). The frontal theta activity in all epochs for both conditions in biathletes and cross-country skiers are shown in Fig 3. For the EVENT, biathletes had on average 6% higher frontal theta activity as compared to cross-country skiers (17.97 ± 1.14 vs 17.07 ± 0.57 μV2 in NOPA and 18.00 ± 1.20 vs 16.90 ± 1.01 μV2 in APA; F1,15 = 4.82, p = 0.044). No significant effect of condition (F1,15 = 0.14, p = 0.72) nor group x condition (F1,15 = 0.25, p = 0.62) on frontal theta activity in the EVENT were found. In the AIM epoch biathletes tended to have on average 6% higher frontal theta activity as compared to cross-country skiers (17.76 ± 1.14 vs 16.87 ± 0.57 μV2 in NOPA and 17.80 ± 1.22 vs 16.70 ± 1.12 μV2 in APA; F1,15 = 4.46, p = 0.052). No significant effect of condition (F1,15 = 0.11, p = 0.74) nor for group x condition (F1,15 = 0.24, p = 0.63) on frontal theta activity in AIM were found. The average theta activity in epoch 5 (Fig 3) was significantly higher than the theta activity during AIM for both groups (F1,31 = 20.45, p < 0.01).

## Discussion

The current study employed EEG to compare frontal theta activity during shooting between biathletes and cross-country skiers and to examine the corresponding effects of vigorous exercise. Coinciding their better shooting performance, biathletes had higher frontal theta activity compared to cross-country skiers for the shooting event (i.e., from 2 seconds before to 1 second after the trigger pull) which confirms our hypothesis that experts have higher frontal theta activity than novices during shooting. Vigorous exercise did not affect shooting performance or frontal theta activity in either biathletes or cross-country skiers. In this case there was no difference between cross-country skiers compared to biathletes, and the expectation that vigorous exercise would decrease shooting performance and frontal theta activity more among cross-country skiers as compared to biathletes was therefore not confirmed. In addition, frontal theta activity during the first 0.5 seconds after the trigger pull was significantly higher than during the aiming period for both groups. Also during this period frontal theta activity was higher among biathletes compared to cross-country skiers in both conditions.

The generally higher frontal theta activity in experts than novices found here is in line with previous findings in golf [2] and shooting [1, 16] and indicates higher focused attention in biathletes. In our study, biathletes had significantly higher frontal theta activity specifically in epoch 3 (i.e., 1 to 0.5 seconds before trigger pull) in NOPA. Doppelmayr et al. [1] also found significantly higher frontal theta activity in expert sharpshooters as compared to novices in the period 1 to 0.5 seconds before trigger pull, and additionally localized this activity to the area near the ACC through source reconstruction (mathematical modelling). These authors concluded that experts are superior in timing their focused attention. This seems to apply also to our findings, although there were tendencies for group differences in frontal theta activity in all epochs. Furthermore, Haufler et al. [16] argued that lower theta activity in novice shooters could be a result of less automatization of the shooting process compared to experts, and suggested that the novice shooters direct their attention to optimize the body-position instead of performance enhancing information.

The lack of a decrease in frontal theta activity and shooting performance after vigorous exercise found in both groups examined in the current study may reflect their highly endurance trained characteristics and subsequent ability to maintain focused attention after physical exercise. It should be noted that the level of exercise was not exhaustive and that the small time lapse between exercise and shooting allowed for some recovery. Still, HR was well elevated above the NOPA level, indicating that physical state of the athletes differed between NOPA and APA. This moderate level of physical stress may well explain the contrast between our results and Baumeister et al. [17], who found a decrease in frontal theta activity and reduced performance in a knee-angle reproduction task in 12 healthy men after exhaustive exercise on a cycle ergometer.

We found no exercise-induced difference in frontal theta activity and shooting performance in neither biathletes nor cross-country skiers. Therefore, vigorous exercise did not cause further decrease in frontal theta activity among cross-country skiers than biathletes, which could be explained by the fact that both groups are trained to sustain high focused attention during endurance competitions. Both biathletes and cross-country skiers regularly compete on high exercise intensities, and even if cross-country skiers do not perform precision tasks such as shooting they may have well-developed skills in maintaining focus during and after exercise. However, it might be more challenging to sustain the high frontal theta activity obtained by biathletes than the lower levels in cross-country skiers after vigorous exercise. Future studies should clarify the role of exercise intensity (including recovery) and the training status in this matter. One option is to include physically untrained expert shooters and compare them with well-trained biathletes to provide further insight into the effect of physical fitness on the ability to maintain high frontal theta activity when exhausted.

The higher frontal theta activity directly after trigger pull compared to the aiming period for both groups could be related to the visual feedback from the target. According to the reinforcement learning theory [27] the ACC is thought to play an important role in error processing and recruits necessary adjustments based on sensory feedback directly through cortico-cortical projections (e.g. with sensorimotor cortex or motor cortex) or indirectly through the striatum and ventral tegmental area [28]. The higher frontal theta activity in epoch 5 in both groups could therefore indicate a response in the ACC due to the feedback after trigger pull. The significantly higher frontal theta activity in biathletes in epoch 5 in both conditions might suggest that biathletes interpret and process the feedback from the target differently than the cross-country skiers, but the mechanisms behind this remain unknown. Previously, significant differences in frontal theta activity between successful and unsuccessful basketball free throws have been documented [29] and the dynamics of theta activity in several brain areas have been related to error processing [30]. Therefore, future research on biathlon could benefit from investigating the impact of feedback from the target by taking away the visual feedback from the target, comparing theta activity between successful and unsuccessful shots and in several brain areas.

### Methodological considerations

The data presented in this study was analysed from surface EEG, which means the underlying neural generators are not directly measured. A reconstruction of the generation of this frontal theta frequency might be possible with source reconstruction based on mathematical modelling or with a co-registration of structural MRI to have an individualized source of specific electrical signals. However, more channels would be preferable (e.g. 128-channel EEG) in future experiments aiming to study activation and localization of frontal brain sources. In addition, more baseline measurements between shooting series would have been preferable, but due to the amount of shots and the long duration of our experiment this was not possible in our case.

We acknowledge that the analyses presented in the current study only provides parts of the overall picture of the various EEG measured factors related to expert performance of a precision task. In this study, we solely focused on theta activity of the frontal cortex which is shown as the main correlate to focused attention [1,8,29]. However, also other brain regions and frequency bands, such as the temporal alpha activity, has been shown to differ between expert and novice performance [16,17,18,19].

### Conclusion

Compared to cross-country skiers with comparable endurance capacity, biathletes had significantly higher frontal theta activity during shooting, indicating a superior ability to focus on the sensory information important for the shooting task in biathletes. Neither biathletes nor cross-country skiers showed an effect of vigorous exercise on shooting performance or frontal theta activity. Therefore, in contrast to what was hypothesised, vigorous exercise did not cause further decrease in frontal theta activity among cross-country skiers than biathletes which could be explained by the fact that both groups are trained to sustain high focused attention during endurance competitions. However, the findings should be interpreted with care and further research looking at connectivity between more brain sources than only the frontal location should help to clarify the underlying neurophysiological mechanisms.
